# Modelling the risk of West Nile virus infection in seven European countries from published serological and case notification data, 2008 to 2022

**DOI:** 10.2807/1560-7917.ES.2026.31.22.2500394

**Published:** 2026-06-04

**Authors:** Hailin Feng, Giovanni Marini, Éva Barabás, Michalis Koureas, Varvara A Mouchtouri, Ilaria Dorigatti

**Affiliations:** 1 MRC Centre for Global Infectious Disease Analysis, School of Public Health, Imperial College London, United Kingdom; 2 Research and Innovation Centre, Fondazione Edmund Mach, San Michele all’Adige, Italy; 3 Hungarian National Blood Transfusion Service, Budapest, Hungary; 4 Laboratory of Hygiene and Epidemiology, Faculty of Medicine, University of Thessaly, Larissa, Greece

**Keywords:** West Nile virus, force of infection, Europe, human, modelling, systematic review

## Abstract

**BACKGROUND:**

West Nile virus (WNV) is a zoonotic mosquito-borne pathogen increasingly reported in Europe.

**AIM:**

We aimed to characterise heterogeneities in the average annual human risk of WNV infection (force of infection, FOI) and in WNV surveillance across Europe.

**METHODS:**

We conducted a systematic review following the PRISMA guidelines to identify serological studies on WNV in humans with IgG-based assays in Europe. We then used mathematical models fitted to both age-stratified serosurvey and case data to reconstruct spatially explicit FOI estimates, the sensitivity of syndromic surveillance and age-dependent trends in case reporting.

**RESULTS:**

We extracted 92 serosurvey datasets from 21 countries. Based on 10 age-stratified serosurvey datasets from Greece, Hungary, Italy, Romania and Spain and case data from seven countries (Austria, Cyprus, Greece, Hungary, Italy, Romania and Spain), we estimated the WNV FOI for 119 European nomenclature of territorial units for statistics level (NUTS) 0-3 regions. We found evidence of spatial heterogeneities in transmission intensity and estimated that on average less than 0.2% of human WNV infections were notified, with country variability and age-dependent trends in the propensity of reporting WNV disease.

**CONCLUSION:**

This study shows that the intensity of WNV transmission, the average annual incidence of infection and the sensitivity of surveillance are heterogeneous across Europe. Due to differences in case reporting across countries, the incidence of reported WNV cases does not necessarily reflect the same proportion of WNV infections and hence the actual infection incidence, which highlights the importance of conducting WNV seroprevalence surveys.

**Key public health message**

**What did you want to address in this study and why?**

West Nile virus (WNV) is transmitted via mosquitoes to humans and other animals. Infections of West Nile virus are increasing in Europe, and the disease has a considerable impact on human health including blood and transplant safety. We aimed to estimate the average annual risk of WNV infection in humans and analyse potential differences in reporting of WNV infections across Europe.

**What have we learnt from this study?**

We identified hotspots of transmission and learnt that the average annual risk of WNV infection and reporting across the 119 European NUTS 0–3 regions included in this study is heterogeneous. Notably, we found that the locations reporting the largest number of WNV reported cases are not necessarily those experiencing the highest number of infections.

**What are the implications of your findings for public health?**

This study suggests that WNV surveillance detects different proportions of the actual (unobserved) number of infections across Europe. Seroprevalence surveys can help estimate the true number of infections, the susceptibility of the population and assess the local transmission risk.

## Introduction

West Nile virus (WNV) is an emerging vector-borne zoonosis mainly transmitted by *Culex* mosquitoes, first isolated in 1937 in Uganda, and now widely spread worldwide, including Europe [1,2]. The virus is maintained in an avian-mosquito transmission cycle and occasionally spills over to humans and horses, which are dead-end hosts [1].

Approximately 20% of patients with WNV infections present acute, influenza-like and self-limiting febrile symptoms (West Nile fever, WNF), and estimates suggest that about 1% of people infected with WNV develop severe neuro-invasive disease (West Nile neuro-invasive disease, WNND) which can be fatal [3]. The incubation period of WNV infection in humans usually ranges from 2 to 14 days but may extend up to 21 days among persons with weakened immune systems [4,5]. In Europe, the transmission season typically lasts between May and October [1,6,7]. Human-to-human transmission can occur through blood transfusion, organ transplant and from mother to baby via breastfeeding or during pregnancy [2,8]. In the absence of a vaccine and antiviral treatment against WNV infection in humans, disease prevention relies on vector control measures and implementation of blood safety protocols. Surveillance of WNV involves monitoring of mosquitoes and other animals (equids and birds) as well as humans [9]. Findings from surveillance activities can inform risk management and the implementation of preventive measures such as vector control interventions. In the European Union (EU) [10], a deferral of at least 28 days for blood donors who travelled to the regions currently recognised to be affected by the virus, such as regions with at least one reported human WNV case, is in place to reduce the risk of human-to-human transmission. In some European countries, such as in Italy, screening for WNV among blood donors is initiated whenever WNV transmission is detected not only through human case surveillance, but also through entomological surveillance, given WNV detection in mosquitoes typically anticipates case detection in humans [11,12].

Due to the clinical presentation of WNV infections, and specifically the development of symptoms and severe neurological conditions in a small proportion of the infections, surveillance of symptomatic human cases gives a partial picture of the extent and occurrence of WNV infections in Europe. On the other hand, detection of IgG antibodies in the blood of infected individuals is a practical way to identify past WNV infections irrespective of the onset of symptoms (if any), estimate the number of infected persons, and by comparing the number of notified cases with the estimated number of infections it is possible to estimate the sensitivity of case-based surveillance. Age-stratified seroprevalence surveys can be used to estimate the force of infection (FOI), defined as the per capita risk at which individuals become infected per unit time (usually expressed in years), which is a key epidemiological metric of transmission intensity [13-16]. Moreover, also the age distribution of notified WNV cases can be used to estimate the FOI, as shown by the methods previously developed for other arboviruses such as dengue virus [17-20] and in combination with age-stratified seroprevalence data provide the information to estimate age-dependent trends in reporting.

In Europe, previous studies modelled spatial and temporal variations in the risk of WNV infection in humans using occurrence data and environmental, land use and economic changes as drivers [17,18,21,22], with some models taking into consideration the avian community composition [23,24]. Mechanistic models have also been employed to reconstruct temporal variations in WNV transmission dynamics [25]. Entomological traits, such as temperature dependencies in mosquito biology, have been included in the calculation of WNV basic reproduction number, R_0_, to predict the impact of climate change [26,27]. Furthermore, the contribution of ecological and climatic factors to WNV establishment across Europe has been quantified using machine learning techniques [28,29].

In this work, we identified and collated data from published WNV serosurveys conducted in Europe and jointly modelled the observed age profile of persons with antibodies against WNV with the age distribution of notified cases to estimate the WNV FOI, age trends in case reporting and the magnitude of case underreporting across Europe. We aimed to characterise spatial variations in the risk of WNV infection and case reporting by linking seroprevalence and case data.

## Methods

### Systematic review

By following the PRISMA guidelines, we conducted a systematic review to identify published serosurveys conducted in Europe of WNV in humans [30]. We used search terms related to “West Nile Virus”, “Europe” and “seroprevalence” in Embase and Medline and restricted the screening to studies conducted in the countries geographically classified as belonging to Europe but without imposing any restriction on language. All search terms are listed in Supplementary List 1. Articles published up to 16 September 2024 (last search date) were retrieved, and for the studies included in the literature review, we also considered the references of the selected papers for possible inclusion.

Three reviewers (HF, GM and ID) were involved in the title and abstract screening and full-text review of the papers, and one reviewer (HF) was responsible for the data extraction. All three reviewers participated in the decision-making process on whether to include the study in the systematic review. The removal of duplicated papers and inclusion decisions were recorded in Covidence (https://www.covidence.org). We applied the following eligibility criteria: (i) the population tested should not include symptomatic WNF/WNND individuals; (ii) the number of positive samples and the total number of samples tested or the estimates of persons with antibodies against WNV (e.g. expressed as percentages) should be provided; (iii) the serological tests used were clearly stated, and these assays quantified IgG antibodies (i.e. studies using assays measuring only IgM antibodies were excluded); (iv) the study location (georeferenced or area-level) was clearly specified. When the included studies mentioned that more granular data compared with the published data were available, we reached out to the authors enquiring for the opportunity to access the more granular data (e.g. the age of the tested persons).

For each eligible study, we collated the following information: year of the study, location (with nomenclature of territorial units for statistics level 3 (NUTS3) code where applicable), serological assay used, total number of persons tested and number of positives or the observed proportion of seropositive persons by age group. If the year of the study was not reported, the publication year was used as a proxy of the year of the study. The database was collated using Microsoft Excel (version 16.89) and the data collected are presented in Supplementary data file 1.

### Regression analysis

To examine the relationship between the assay used and the reported overall seropositivity (*π_i_*) in each study (*i*), we ran a regression analysis between *π_i_* and the serological assay used (*X*_i_) (reference category: neutralisation test) adjusted for the study period (*T_i_*, defined as 16-year intervals starting from 1958 up to 2021, reference group: 1958–1973) as shown in Equation (Eqn.) (1) where ε is a normally distributed error term. For the age-stratified seroprevalence survey data, we reconstructed the overall population-level seropositivity by weighting the age-specific seropositivity by the corresponding age-distribution of the population [31].

$\pi_{i}=\beta_{0}+\beta_{1}X_{i}+\beta_{2}T_{i}+\varepsilon \begin{array}{cc} & \left(1\right) \end{array}$

### Model parameterisation and fitting

#### Matching serosurvey and case data

We obtained WNV epidemiological data collected between 2008 and 2022 from the European Surveillance System (TESSy) provided by Austria, Bulgaria, Croatia, Cyprus, Czechia, France, Germany, Greece, Hungary, Italy, the Netherlands, Portugal, Romania, Slovakia, Slovenia and Spain and released by the European Centre for Disease Prevention and Control (ECDC), by NUTS3 regions. As the EU WNV case definition was standardised in 2008, we included cases reported starting from that year in our analysis [32-35]. We extracted the annual number of reported cases in TESSy by age groups of 10-year intervals (e.g. 0–9 years, 10–19 years, up to age group ≥ 90 years) at NUTS3 level.

In the subsequent modelling analysis, we retained only regions with WNV serosurveys conducted since 2008 in the general population, including blood donors, and that reported seropositivity in at least two age groups. We then matched the age-stratified serosurvey data to the case data reported in TESSy at NUTS3 level. Regions with fewer than 10 reported cases between 2008 and 2022 and without any age-stratified seroprevalence data available were excluded from the modelling analysis.

#### Region classification

We classified the NUTS3 regions with serosurvey and/or WNV case data into three categories: (i) regions with age-stratified seroprevalence data only; (ii) regions with case data only; (iii) regions with both age-stratified seroprevalence and case data. We denote *λ_l_* the location-specific and time-constant WNV FOI, i.e. the per capita average annual probability for susceptible people living in location *l* of acquiring WNV infection, which we estimated on the logarithmic scale using the mathematical models developed for each region category.

#### Model for regions with age-stratified seroprevalence data only

We respectively defined *N^l^_j_* as the number of tested samples and and *P^l^_j_* as the number of positive samples in location *l* and in age group *j* with age group median *a_j_*. Using an established catalytic model [14,16,36,37], the seroprevalence *π^l^_j_* of age group *j* in location *l*, was modelled as a function of the local WNV FOI *λ_l_* as described in Eqn. (2): 

$\pi_{j}^{l}=1-e^{-\lambda_{l}a_{j}}\begin{array}{cc} & \left(2\right) \end{array}$

For each location, we assumed that the observed data followed a binomial likelihood as defined in Eqn. (3): 

$L(P^{l},N^{l}|\lambda_{l})=\prod_{j}{\pi_{j}^{l}}_{}^{P_{j}^{l}}{\left(1-\pi_{j}^{l}\right)}^{N_{j}^{l}P_{j}^{l}}\begin{array}{cc} & \left(3\right) \end{array}$

#### Model for regions with reported case data only

In regions with only case data, we estimated the location-specific FOI (*λ_l_*), the yearly country-specific reporting rate (*ρ_c_*) for country *c* and the age-dependent trend in case reporting (*γ_j_*) by using the model [13,17,19,20] described in Eqn. (4): 

$C_{j}^{l}=\rho_{c}\gamma_{j}S_{j}^{l}\left(e^{-\lambda_{l}a_{j}^{\min}}-e^{-\lambda_{l}a_{j}^{\max}}\right)\begin{array}{cc} & \left(4\right) \end{array}$

In this model, *C^l^_j_* denotes the expected number of reported cases during the study period (2008–2022), *S^l^_j_* denotes the average population size of age group *j* in location *l *[38] multiplied by the length of study period (15 years), and *a_j_^min^* and *a_j_^max^* denoted the lower and upper bound of age group *j*. We estimated the age-dependent trends in case reporting *γ_j_* (for *j* ≠ 8) relative to the reference age group of 70–79-year-olds (*γ_8_* = 1). We assumed that the observed age group specific number of reported cases in location *l*, *O^l^_j_*, follows a multinomial distribution as described in Eqn. (5), and that the observed total number of reported cases in location *l*, *O^l^*, follows a Poisson distribution as defined in Eqn. (6).

$L(O_{j}^{l}|C_{j}^{l})=\prod_{j}{\left(\frac{C_{j}^{l}}{\sum_{j}C_{j}^{l}}\right)}^{O_{j}^{l}}\begin{array}{cc} & \left(5\right) \end{array}$

$L(O^{l}|\sum_{j}C_{j}^{l})=\prod_{l}\frac{{\left(\sum_{j}C_{j}^{l}\right)}^{O_{l}}}{O_{l}!}e^{-\sum_{j}C_{j}^{l}}\begin{array}{cc} & \left(6\right) \end{array}$

#### Model for regions with both age-stratified seroprevalence and case data

In regions with both age-stratified seroprevalence and case data, we estimated the location-specific FOI *λ_l_*, the yearly country-specific reporting rate (*ρ_c_*) and the age-dependent trend in case reporting (*γ_j_*, reference age group of 70–79-year-olds, *γ_8_* = 1) as described in Eqn. (2)-(6).

#### Prior distributions

We used normal prior distributions centred in −7 and with standard deviation 3.5 for all location-specific FOIs estimated in the log scale (ln(*λ_l_*)). For the country-specific reporting rates (*ρ_c_*), we used normal prior distributions, centred in the raw reporting rate estimates computed as the ratio between the number of reported cases and the local total number of seropositive individuals in the population during the study period reconstructed from the serosurvey data. The standard deviation of the prior distribution of *ρ_c_* was set to the value calculated over all countries.

For each age group *j* (except *j* = 8 which was taken as reference group), we used normal prior distribution for *γ_j_* centred on the mean proportion of cases reported in the average annual age-specific population *S̅_J _*relative to the proportion of annual cases reported in the reference age group, 70–79-year-olds, as shown in Eqn. (7). 

${\hat{\gamma }}_{j}=\frac{O_{j}/{\bar{S}}_{j}}{O_{8}/{\bar{S}}_{8}}\begin{array}{cc} & \left(7\right) \end{array}$

The prior distribution of all parameters is shown in Supplementary Table 1.

#### Model fitting

We jointly fitted the model to all NUTS3 regions with both seroprevalence and case data and only case data using the Hamilton Monte Carlo (HMC) method implemented in *CmdStanR* [39]. The regions with only age-stratified seroprevalence data were fitted separately, given that the only estimated parameter was *λ_l_*.

We ran 25,000 iterations of the HMC method in three parallel chains, using a burn-in of 7,000 iterations and a thinning of five iterations. In total, we generated 15,000 samples from the posterior distributions which we then used to estimate the median and 95% credible interval (CrI) of the parameters.

### Incidence estimation

We estimated the average annual number of new WNV infections (*Ĉ_l_*) for each location using the estimated FOI (*λ̂_l_*) and the average annual population size (*S̄^l^_j_*) at NUTS3 level as shown in Eqn. (8).

${\hat{C}}_{l}=\sum_{j}{\bar{S}}_{j}^{l}\left(e^{-{\hat{\lambda }}_{l}a_{j}^{\min}}-e^{-{\hat{\lambda }}_{l}a_{j}^{\max}}\right)\begin{array}{cc} & \left(8\right) \end{array}$

### Statistical analysis

We tested whether the FOI estimates obtained in the regions with age-stratified seroprevalence data were significantly different in mean from the FOI estimates obtained in the regions with age-stratified case data only using the Welch two sample t-test.

### Sensitivity analysis

We conducted a sensitivity analysis to assess the effect of accounting for both data sources (age-stratified seroprevalence data in combination with age-stratified case data) vs only considering age-stratified case notification data or only age-stratified seroprevalence data, on the FOI estimates. The comparison between estimated parameters was only conducted on the NUTS3 regions with both age-stratified serosurvey and case data. We estimated the FOI using Eqn. (2)–(3) (only serosurvey data), Eqn. (4)–(6) (only case notification data) and compared these with the FOI estimates obtained using Eqn. (2) to (6) (both serosurvey and case data).

## Results

### Systematic literature review

**Figure 1:**
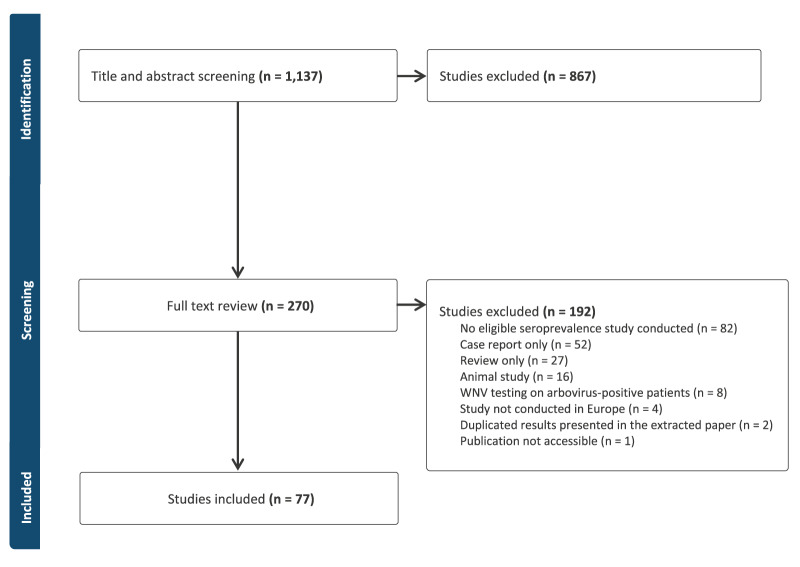
PRISMA chart of the systematic review on serosurveys of West Nile virus in humans, Europe, 1958–2021^a^

^a^ Studies with publication date until 16 September 2024 were included.

Figure 1 shows the PRISMA chart of the literature review, which included 1,137 published studies: 270 were retained for full text review. We extracted data from 77 studies; these provided 92 serological datasets for 233 NUTS1–3 administrative units or geolocations in 21 European countries, as presented in Supplementary data file 1 and Supplementary Figure 1. In addition, we analysed previously unpublished age-stratified data from seroprevalence surveys conducted in Greece and Hungary, which were originally published at an aggregated level [40,41]. We identified 18 age-stratified datasets, while all other 74 datasets reported the overall (i.e. overall ages, not stratified by age group) proportion of seropositive people.

The serosurveys included in this review were conducted between 1958 and 2021. For 18 of the 92 datasets, we used the publication year as a proxy for the year of the study. The study population ranged from healthy blood donors, residents and international travellers, and specific populations screened for occupational health, such as zoo, forestry and stable workers, farmers and hunters. The number of persons tested in the serosurveys included varied from seven in Italy in 2010 [42] to 14,437 in Germany in 2004 [43].

The number of serosurveys, assays used and the overall reported proportion of seropositive persons varied both temporally and spatially, as can be seen in Supplementary Figure 2. Various serological assays were used across serosurveys, including ELISAs, immunofluorescence assays (IFAs), hemagglutination-inhibition tests (HITs), neutralisation tests and protein microarray. For 50 of the 92 serosurveys, multiple serological tests were conducted on the same samples to assess their concordance and sensitivity and potential cross-reactivity with other viruses [41]. Neutralisation test was used throughout the study periods and appeared in most combinations with other serology tests, while the IFA was mainly used in combination with other assays; HIT was mainly used before 1989 while ELISA has been used frequently since 1990s. Protein microarray was used in only one study [44]. The regression analysis showed that compared with the neutralisation test, HIT was associated with significantly higher overall seropositivity, after adjusting for the period (p = 0.015). More details are provided in Supplementary Figure 2B and Supplementary Table 2.

The 18 age-stratified seroprevalence datasets were extracted from 17 serosurvey studies conducted between 1963 and 2020, with sample sizes ranging from 65 to 3,962 participants (Table). All serosurveys were conducted in eastern and southern Europe, except for one study in Austria and one in Germany. We used 10 age-stratified seroprevalence surveys done in 2008–2022 for the FOI estimation. In eight of these studies, neutralisation tests were used, and in two studies, the samples were tested for other cross-reactive arboviruses, with the cross-reactive samples removed from the analysis. Two studies were national serosurveys conducted in Hungary, seven studies covered 58 NUTS3 regions in Greece, Spain, Italy and Romania, and one study covered a NUTS2 region in Greece.

**Table:** Age-stratified serosurvey datasets identified in a systematic review on West Nile virus infection in humans, by year, Europe, 1963−2020^a^

Publication	Country	Area	Target population	Sampling method	Study year	Sample size
Kunz [55]	Austria	Klosterneuburg	Blood donors	Convenience sampling	1963^b^	65
Pavlatos et al. [56]	Greece	National	General population	Probability proportional to size sampling	1964^b^	1,128
Ebke et al. [57]	Germany	National (except Schleswig-Holstein)	General population	Convenience sampling	1971^b^	373
Garea Gonzalez et al. [58]	Spain	North-western Spain	General population	Convenience sampling	1977^b^	701
Imami et al. [59]	Kosovo^c^	Suhareka	General population	Convenience sampling	1978^b^	992
Hubalek et al. [60]	Czechia	Prague	General population	Voluntary sampling	2002, 2003	497
Dimou et al. [61]	Greece	Northern Greece	General population	Convenience sampling	2003–2005	655
Bernabeu-Wittel et al. [62]	Spain	Seville	General population	Stratified random sampling	2007^b^	504
Ladbury et al. [51]^a^	Greece	Lowland areas of Imathia and Pella prefectures	General population	Two stage cluster sampling	2010	644
Remoli et al. [63]^a^	Italy	Grosseto	General population	Convenience voluntary sampling	2012	100
Hadjichristodoulou et al. [40]^a, d^	Greece	National	General population	Stratified left-over sampling	2012–2013	3,962
Nagy et al. [41]^a, d^	Hungary	National	Blood donors	Random sampling	2016	2,112
Marchi et al. [52]^a^	Italy	Siena	General population	Convenience sampling	2016–2017	879
Frías et al. [64]^a^	Spain	Ciudad Real	Blood donors	Disproportionate stratified random sampling	2017–2018	1,093
Marchi et al. [52]^a^	Italy	Siena	General population	Convenience sampling	2018–2019	921
Crivei et al. [65]^a^	Romania	Iasi	Hospital-based cohort	Convenience sampling	2019	88
Nagy et al. [66]^a^	Hungary	National	Blood donors	Convenience sampling	2019–2020	3,005
Coroian et al. [67]^a^	Romania	Alba, Cluj, Sălaj, Bistrița-Năsăud, Maramureș and Satu Mare	Blood donors	Disproportionate stratified sampling	2019–2020	1,200

^a^ Only the studies conducted from 2008 (n = 10) were included in the FOI estimation.

^b^ The year of publication was used as a proxy for the year of the study.

^c^ At the time of the published study [59] Kosovo was part of Yugoslavia. The geographical designation Kosovo is without prejudice to positions on status and is in line with United Nations Security Council Resolution 1244/99 and the International Court of Justice Opinion on the Kosovo declaration of independence.

^d^ This study provided more disaggregated data than originally published.

### Region categorisation

In total, we found 31 NUTS3 regions (in Greece, Spain, Italy, Bulgaria, France and Romania) with evidence of WNV circulation from serosurveys but no previously reported cases in TESSy between 2008 and 2022. During the study period, 4,460 WNV human cases were reported in 200 NUTS3 regions in TESSy.

We estimated the WNV FOI for 119 regions across seven countries. These included 19 NUTS2 and NUTS3 regions from four countries (Greece, Italy, Romania and Spain) and a national estimate from Hungary with only age-stratified seroprevalence data, 40 NUTS3 regions with both age-stratified seroprevalence data and case data from two countries (Greece and Romania), and 59 regions with only case data (≥ 10 cases) from six countries (Austria, Cyprus, Greece, Hungary, Romania and Spain) reported in 2008–2022. For completeness, we show the NUTS3 regions with < 10 reported cases between 2008 and 2022 and without any age-stratified seroprevalence data available (hence excluded from the FOI analysis) in Supplementary Figure 3.

**Figure 2:**
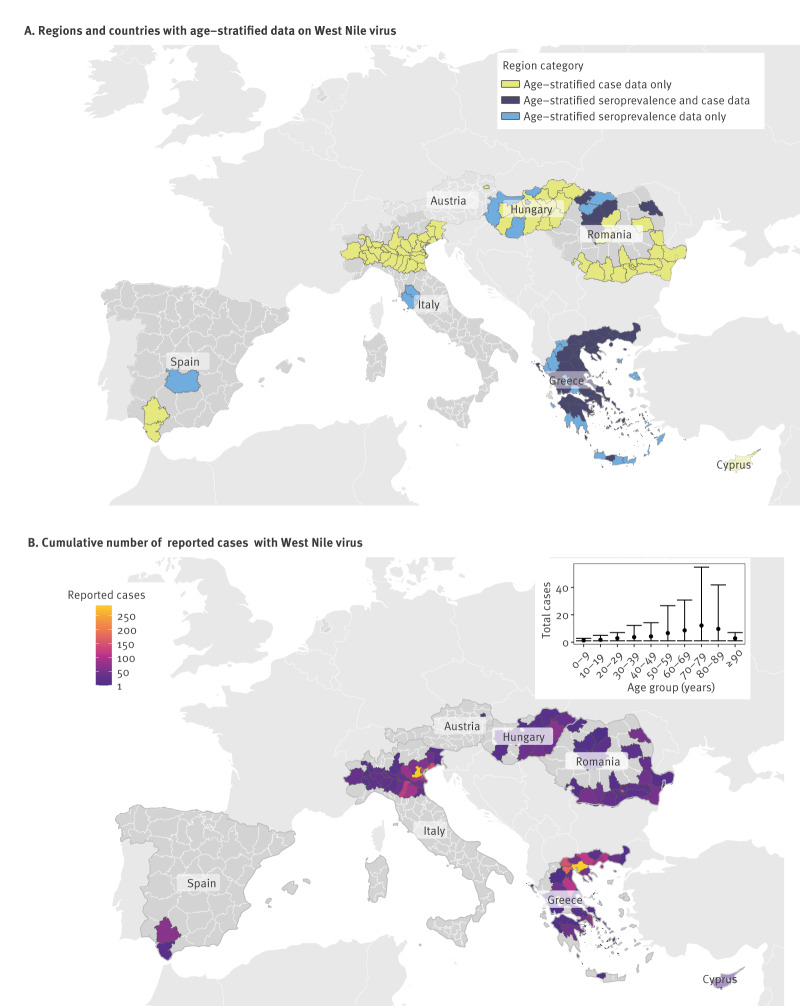
Regions with West Nile virus infections in humans being modelled, seven European countries, 2008–2022

ECDC: European Centre for Disease Prevention and Control; EU/EEA: European Union/European Economic Area; NUTS: Nomenclature of Territorial Units for Statistics.

Figure 2A shows the locations of the regions with available WNV data analysed in this study, and Figure 2B shows the cumulative number of cases reported by NUTS3 region where the inset shows the combined age-distribution of the cases over all location, with the highest incidence in persons aged 70–79 years.

Panel A. The regions are shaded with colours representing the region category. For regions with only case data, only regions with ≥ 10 cases reported over the study period are shown.

Panel B. Cumulative number of cases (over all ages) reported between 2008 and 2022 in 99 NUTS3 regions included in the FOI modelling. Data provided by ECDC based on the data reported by EU/EEA countries. The inset shows the age group distribution calculated over all locations. The points show the mean of cumulative reported cases during the study period by age group calculated over the NUTS3 regions and the bars indicate the 2.5% and 97.5% percentiles.

### Prior distributions

The prior distributions of the reporting rates of Supplementary Table 1 were informed by the raw reporting rates estimates by country (Austria, Cyprus, Greece, Hungary, Italy, Romania, Spain), as presented in Supplementary Table 1 and Supplementary Figure 4. Overall, we estimated that on average 0.1% of the seropositive population are reported in the case-based surveillance, with a standard deviation of 0.17%. The propensity of case reporting shows an increasing trend with age, Supplementary Figure 5.

### Model fit

**Figure 3:**
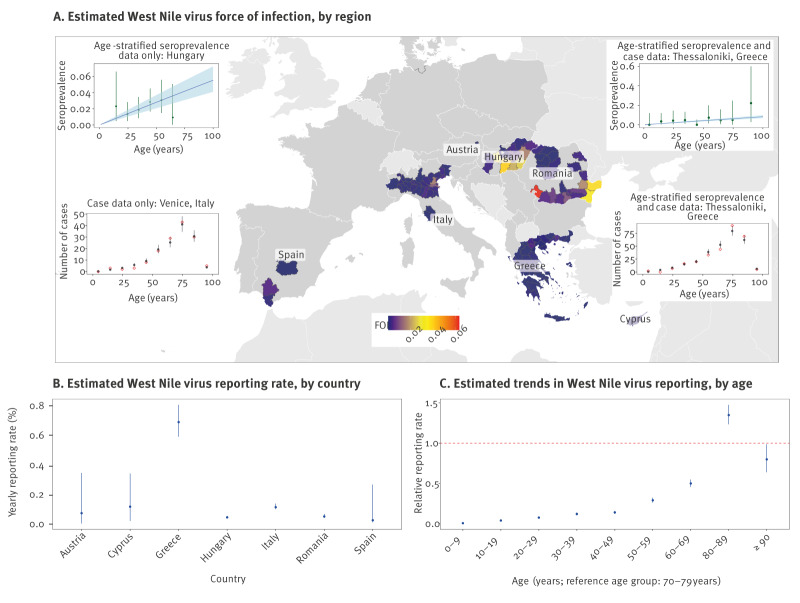
Estimated force of infection, reporting rate and trends in reporting by age, seven European countries, 2008–2022

CrI: credible interval; FOI: force of infection; WNV: West Nile virus.

We included age-stratified seroprevalence survey and case data across 119 regions from Austria, Cyprus, Greece, Hungary, Italy, Romania and Spain in the analysis. More detailed information is presented in Supplementary Figure 6 and 7. Figure 3 shows the FOI estimates, the yearly country-specific reporting rates and the age-dependent trend in case reporting, including examples of the model fit for (i) the metropolitan city of Venice (Italy), for which we only had case data; (ii) Thessaloniki region (Greece), for which we had both age-stratified seroprevalence and case data and (iii) Hungary, for which we only had national age-stratified serosurvey data. The results presented in Figure 3A suggest large spatial heterogeneity in the estimated WNV FOI, from 1.44 × 10^−5^ (95% CrI: 1.40 × 10^−6^–5.89 × 10^−5^) in Magnesia, Greece to 6.32 × 10^−2^ (95% CrI: 4.49 × 10^−2^–8.39 × 10^−2^) in Olt, Romania. Hotspots of WNV transmission, defined as regions with median estimates of the FOI > 0.033 (corresponding to an average age of infection at 30 years), were identified in Romania and Hungary. The FOI estimates obtained for the regions reporting case data only tended to be significantly higher (p < 0.001) than the FOI estimates obtained for the regions with age-stratified seroprevalence data, as presented in Supplementary Figure 8.

Panel A. Median FOI estimates at regional level from seven European countries. The FOI estimates at NUTS3 level are shaded on top of the NUTS2 estimate where applicable. The insets show three examples of each region category: Hungary (national age-stratified serosurvey data only); city of Venice (Italy) (case data only); Thessaloniki region (Greece) (both age-stratified case and seroprevalence data available). The observed serosurvey data (point) and 95% confidence interval calculated using the exact binomial method (error bar) are shown in green; the blue line and shaded area show the median and 95% CrI of the model estimates. The observed number of reported cases are shown in red; black points and vertical lines show the median and the 95% CrI of the model estimates.

Panel B. Estimated yearly country-specific reporting rate (median and 95% CrI).

Panel C. Estimated age-dependent trend in reporting a WNV case compared with the reference age group of 70–79-year-olds (median and 95% CrI).

As shown in Figure 3B, we estimated that case-based surveillance reports around 0.69% (95% CrI: 0.59–0.80%) of WNV infections in Greece, 0.12% (95% CrI: 0.02–0.34%) in Cyprus, 0.11% (95% CrI: 0.10–0.14%) in Italy and smaller proportions of WNV infections in the other countries. The large uncertainty around the reporting rate estimates in Cyprus, Austria and Spain is because Cyprus is classified as a single NUTS3 region while only two regions in Spain and one in Austria were available for the analysis.

We also estimated the relative propensity of WNV reporting by age group and found increasing trends by age (Figure 3C). Compared with the reference group of 70–79-year-olds, WNV infections tended to be reported 1.35 times more (95% CrI: 1.23–1.47) among 80–89-year-olds, 0.80 (95% CrI: 0.64–0.99) times among persons aged ≥ 90 years and significantly less among younger persons.

**Figure 4:**
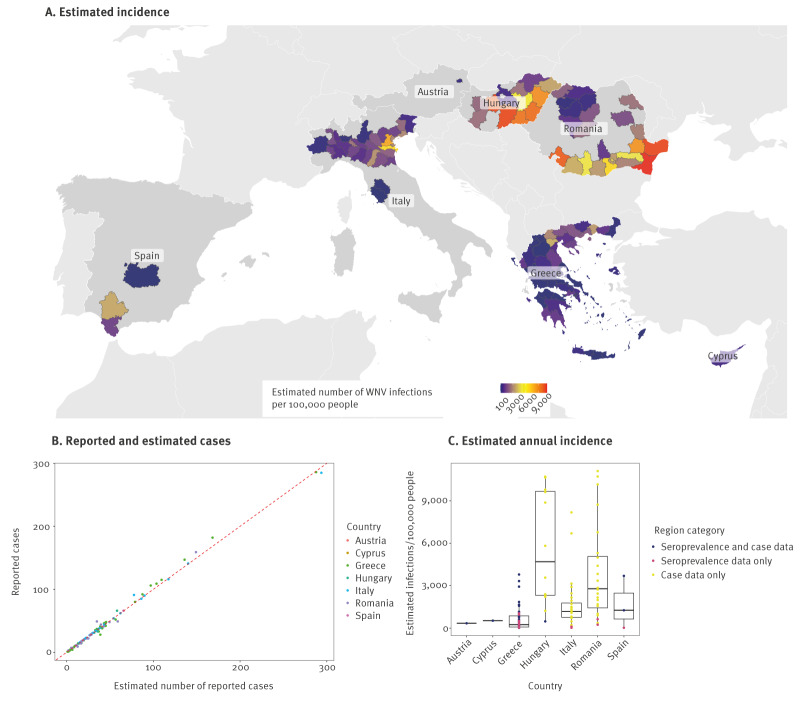
Estimated average annual incidence of West Nile virus infections per 100,000 people and estimated annual number of cases, seven European countries, 2008–2022

FOI: force of infection; NUTS: Nomenclature of Territorial Units for Statistics; WNV: West Nile virus.

Finally, from the annual expected number of WNV infections per 100,000 people extrapolated from the FOI estimates at NUTS3 level, we found hotspots of WNV transmission and infection in large parts of Hungary and Romania and in a few provinces in northern Italy as well (Figure 4). Figure 4B shows that the estimated number of cases from the fitted model at NUTS3 level is in line with the observed number of reported cases. In some NUTS3 regions in Romania, Hungary and Italy, we estimated that the annual incidence of WNV infection, which includes asymptomatic and symptomatic infections, could exceed > 6,000 per 100,000 people (these regions reported case data only; Figure 4C). On the other hand, we estimated that none of NUTS3 regions in Greece had more than 4,000 WNV infections per 100,000 people per year despite parts of Greece being among those reporting the largest number of WNV cases in Europe (Figure 2B). Large within-country variations in the incidence of WNV infection were estimated in Hungary and Romania (Figure 4A).

Panel A. A total of 117 NUTS3 regions included in the estimations. Expected average annual incidence computed using the median FOI estimates from the fitted model.

Panel B. Reported vs estimated cases in 99 NUTS3 eligible regions with data on reported cases. The country of each region is indicated by the colour. The red dashed line shows the y = x line.

Panel C. Boxplot of estimated annual incidence in 117 NUTS3 regions fitted to the model (points) by country and coloured by region category. The box shows the first and the third quartiles and the bold horizontal line shows the median.

### Sensitivity analysis

Among the 40 NUTS3 regions with both age-stratified seroprevalence and case data available, we found that for 32 NUTS3 regions, the FOI estimates obtained were consistent (i.e., had overlapping 95% CrI) when considering only seroprevalence, case data or both data sources, as shown in Supplementary Figure 9A. The remaining eight regions were mainly characterised by large numbers of reported cases (the average number of cases is 90 across the eight regions, with a maximum of 182), suggesting that the case component of the model influenced the model calibration and the FOI estimates the most. Despite the differences in FOI estimates, the estimates of reporting rate and age-dependent scaling factor, showed overlapping 95% CrI when fitting only to case data as illustrated in Supplementary Figures 9B, 9C, 10 and 11 indicating a qualitatively similar model fit to when using both data sources.

## Discussion

In the systematic literature review, we found large variations in the sample sizes, assays used, geographical scope and study populations in the serosurveys conducted in Europe over time. The relatively high WNV seroprevalence before 1990 appears to be linked to the serological assays used. We also found more serosurveys conducted in Europe since 2006, which is likely driven by the increased transmission and upsurge of WNV outbreaks, alongside the strengthening of arbovirus surveillance across the regions [45-47].

Seroprevalence studies help unveil the population exposure to WNV irrespective of the strength of the local surveillance system, as they can estimate the number of persons infected and identify regions with evidence of undetected WNV circulation. For instance, the seroprevalence study conducted in Greece in 2013 identified WNV seropositive persons in seven prefectures where no cases were reported in previous years [40]. One of these prefectures reported WNV cases in 2018 and 2022.

While flavivirus cross-reactivity can pose uncertainties in the interpretation of WNV seroprevalence data [48], in this study, we have adopted stringent selection criteria – including using the results obtained with the neutralisation assay for eight of the 10 age-stratified surveys, and the exclusion of any cross-reactive sample in the remaining serosurveys – which ensures the specificity and sensitivity of the data included in the modelling analysis.

In this study, we adapted catalytic models originally developed to analyse surveillance data from endemic, immunising vector-borne diseases, and largely applied to dengue seroprevalence studies [15,16] and case data [13,19,20], to estimate the WNV FOI from WNV serosurveys and case notification data in Europe. Specifically, sero-catalytic models exploit observed age trends in seroprevalence to infer the risk of infection, as increasing seroprevalence by age indicates endemic viral circulation (given the probability of having acquired the infection depends on the exposure time, measured in years of life, as opposed to flatter age-dependent seroprevalence profiles which indicate equal susceptibility, exposure and risk of infection by age which is typical of epidemic and emerging settings). A summary of the mathematics behind the use of observed age trends in seroprevalence to characterise the level of endemicity and transmission intensity is given in the published literature [37]. In particular, a strength of this study is in the identification of locations with both age-stratified serosurvey and case data in 2008–2022, which provides information and power to estimate the proportion of infections reported to surveillance.

Notably, the estimated variation in the country-specific reporting rate of WNV not only reflects different sensitivities of case-based surveillance but also captures differences in case definitions, as some countries report only confirmed cases [49] and others report also probable cases [35] and, in this study, we analysed probable and confirmed cases combined. On the other hand, in all EU/EEA countries except Czechia, Greece and Spain, passive WNV surveillance was in place, hence WNND are more frequently detected than WNF cases [6]. While the confirmed WNF cases may be younger than the WNND cases, the extent to which geographical differences in the relative proportion of reported WNND vs WNF cases may influence the FOI estimate cannot be extensively evaluated to date, as the breakdown of the total number of reported WNV cases into WNND, WNF or asymptomatic cases was not available. In the future, it would be useful to access the breakdown of WNV cases into WNND, WNF and asymptomatic cases, as this would shed light on differences in surveillance and allow us to test whether applying the methods developed in this study solely on confirmed WNND cases modifies the FOI estimates obtained in this study using all reported WNV cases. In turn, the relative reporting rates increasing with age suggest that older adults are more likely to be symptomatic, develop severe disease and thus seek medical care when infected, which is in line with the published literature [50].

Our FOI estimates suggest that in some European countries, the regions reporting the largest number of cases are not necessarily those where we expect the largest number of infections. In the sensitivity analysis that we have conducted, we find that the FOI estimates obtained using only case data, only serosurvey data and both data sources were consistent. While this finding is reassuring, both from a modelling and public health perspective, it also highlights the benefit of validating and refining FOI estimates using multiple tools and data sources. Specifically, the implementation of regional age-stratified WNV serosurveys across Europe in locations with known WNV circulation generates the data needed to directly quantify the sensitivity of local surveillance which would eventually enable more precise and less uncertain estimates of WNV transmission intensity. Furthermore, serosurveys conducted in regions at risk of WNV transmission provide useful data to better understand the current and future geographical limits of WNV circulation, as the virus is likely to move into new regions characterised by naïve host and human populations before its circulation is detected through WNV surveillance as is currently implemented across most of Europe.

The objective of this study was to quantify the average transmission intensity of WNV, as measured by the time-constant FOI. Due to the limited amount of WNV data available in time from both case-based surveillance and serosurveys, it was not possible to estimate the seasonality nor intra-annual variations in transmission intensity. Sequential serosurveys have been conducted in Greece and Italy [40,51,52], but the small sample sizes in serosurveys posed limitations to the extent to which it was possible to reconstruct temporal changes in the local WNV FOI without substantial uncertainty in the estimates. Despite this limitation, the quantification of spatial variations in the average time-constant FOI helps quantify the (unobserved) burden of infection and can contribute to identifying climate and environmental risk factors [53]. This study also highlights the heterogeneous implementation of population-based age-stratified serosurveys across Europe. The estimates generated in this study agree with previous analyses suggesting parts of eastern Europe and parts of southern Europe such as Italy are at higher risk of WNV transmission [28,29]. In the future it would be interesting to assess the extent to which ecological and climatic factors can explain the variation in the WNV FOI estimates generated in this study [17,28,29,54].

## Conclusion

In conclusion, we developed data fusion modelling methods utilising both serosurvey and case notification data for quantifying the local risk of WNV transmission as determined by the FOI, which accounted surveillance across geographies and age-dependent trends in reporting. Our results suggest that the European regions with the highest incidence of reported WNV cases are not necessarily the regions with the highest transmission risk. As such, this study lays the foundations for informing the development of evidence-based surveillance programmes to inform public health policy, epidemic preparedness and outbreak response planning now and in the future.

## Data Availability

The epidemiological data that support the findings of this study are available from ECDC. Restrictions apply to the availability of these data, which were used under license for the current study, and so are not publicly available. The serosurvey data extracted from the systematic literature review are provided in the Supplementary data file ‘Review spreadsheet.xlsx’.

## Disclaimer

This article has obtained approval of publication from ECDC. The views and opinions of the authors expressed herein do not necessarily state or reflect those of ECDC nor of the Vaccine Impact Modelling Consortium. The accuracy of the authors’ statistical analysis and the findings they report are not the responsibility of ECDC. ECDC is not responsible for conclusions or opinions drawn from the data provided. ECDC is not responsible for the correctness of the data and for data management, data merging and data collation after provision of the data. ECDC shall not be held liable for improper or incorrect use of the data.

## Supplementary Data

5831000 Supplementary Material 1

## Supplementary Data

89000 Supplementary Material 2
