# Supplementary material for: Modelling the risk of West Nile virus infection in seven European countries from published serological and case notification data, 2008 to 2022
Source: Euro Surveill. 2026 Jun 4;31(22):2500394. doi: 10.2807/1560-7917.ES.2026.31.22.2500394 (PMC13330735; doi:10.2807/1560-7917.ES.2026.31.22.2500394)
Supplement: Supplementary Material 1 [file 25-00394_DORIGATTI_Supplement1.pdf]

Supplementary Information

Modelling the risk of West Nile virus infection in seven European countries from published serological and case notification data, 2008 to 2022

Hailin Feng[1], Giovanni Marini[2], Éva Barabás[3], Michalis Koureas[4], Varvara A Mouchtouri[4], Ilaria Dorigatti[1]

1. MRC Centre for Global Infectious Disease Analysis, School of Public Health, Imperial College London, United Kingdom
2. Research and Innovation Centre, Fondazione Edmund Mach, San Michele all'Adige, Italy
3. Hungarian National Blood Transfusion Service, Budapest, Hungary
4. Laboratory of Hygiene and Epidemiology, Faculty of Medicine, University of Thessaly, Larissa, Greece

This supplementary information is hosted by Eurosurveillance as supporting information alongside the article "Modelling the risk of West Nile virus infection in seven European countries from published serological and case notification data, 2008 to 2022", on behalf of the authors, who remain responsible for the accuracy and appropriateness of the content. The same standards for ethics, copyright, attributions and permissions as for the article apply. Supplements are not edited by *Eurosurveillance* and the journal is not responsible for the maintenance of any links or email addresses provided therein.

Table of Contents

LITERATURE REVIEW SEARCH LIST 3

SUPPLEMENTARY TABLES 6

SUPPLEMENTARY FIGURES 8

Literature Review Search List

Supplementary List 1 Keywords for searching literature in databases
Embase search keywords

1. sero*.mp.
2. Antibod*.mp. or exp antibody/
3. prevalence.mp. or exp prevalence/
4. West Nile Virus.mp. or West Nile virus/
5. West Nile fever.mp. or West Nile Fever/
6. West Nile neuroinvasive disease.mp.
7. (West Nile adj2 disease).mp.
8. West Nile Flavivirus.mp.
9. WN virus.mp.
10. West Nile.mp.
11. Europe.mp. or exp Europe/
12. Germany.mp.
13. France.mp.
14. Italy.mp.
15. Spain.mp.
16. Ukraine.mp.
17. Poland.mp.
18. Romania.mp.
19. Netherlands.mp.
20. Belgium.mp.
21. Czechia.mp.
22. Greece.mp.
23. Portugal.mp.
24. Sweden.mp.
25. Hungary.mp.
26. Belarus.mp.
27. Austria.mp.
28. Serbia.mp.
29. Switzerland.mp.
30. Bulgaria.mp.
31. Denmark.mp.
32. Finland.mp.
33. Slovakia.mp.
34. Norway.mp.
35. Ireland.mp.
36. Croatia.mp.
37. Moldova.mp.
38. (Bosnia and Herzegovina).mp.
39. Albania.mp.
40. Lithuania.mp.
41. North Macedonia.mp.
42. Slovenia.mp.
43. Latvia.mp.
44. Kosovo.mp.
45. Estonia.mp.
46. Montenegro.mp.

47. Luxembourg.mp.
48. Malta.mp.
49. Iceland.mp.
50. Andorra.mp.
51. Monaco.mp.
52. Liechtenstein.mp.
53. San Marino.mp.
54. Holy See.mp.
55. Czech Republic.mp.
56. Bosnia.mp.
57. Vatican City.mp.
58. Slovak Republic.mp.
59. Cyprus.mp. or exp Cyprus/
60. 11 or 12 or 13 or 14 or 15 or 16 or 17 or 18 or 19 or 20 or 21 or 22 or 23 or 24 or 25 or 26 or 27 or 28 or 29 or 30 or 31 or 32 or 33 or 34 or 35 or 36 or 37 or 38 or 39 or 40 or 41 or 42 or 43 or 44 or 45 or 46 or 47 or 48 or 49 or 50 or 51 or 52 or 53 or 54 or 55 or 56 or 57 or 58 or 59
61. 1 or 2 or 3
62. 4 or 5 or 6 or 7 or 8 or 9 or 10
63. 60 and 61 and 62

MEDLINE search keywords

1. sero*.mp.
2. (Antibodies or Antibody).mp. or Antibodies/
3. Prevalence/ or prevalence.mp.
4. West Nile Virus.mp. or West Nile virus/
5. West Nile fever.mp. or West Nile Fever/
6. West Nile neuroinvasive disease.mp.
7. (West Nile adj2 disease).mp.
8. West Nile.mp.
9. WN Virus.mp.
10. exp Europe/ or Europe.mp.
11. Germany.mp.
12. France.mp.
13. Italy.mp.
14. Spain.mp.
15. Ukraine.mp.
16. Poland.mp.
17. Romania.mp.
18. Netherlands.mp.
19. Belgium.mp.
20. Czechia.mp.
21. Greece.mp.
22. Portugal.mp.
23. Sweden.mp.
24. Hungary.mp.
25. Belarus.mp.

26. Austria.mp.
27. Serbia.mp.
28. Switzerland.mp.
29. Bulgaria.mp.
30. Denmark.mp.
31. Finland.mp.
32. Slovakia.mp.
33. Norway.mp.
34. Ireland.mp.
35. Croatia.mp.
36. Moldova.mp.
37. (Bosnia and Herzegovina).mp.
38. Albania.mp.
39. Lithuania.mp.
40. North Macedonia.mp.
41. Slovenia.mp.
42. Latvia.mp.
43. Kosovo.mp.
44. Estonia.mp.
45. Montenegro.mp.
46. Luxembourg.mp.
47. Malta.mp.
48. Iceland.mp.
49. Andorra.mp.
50. Monaco.mp.
51. Liechtenstein.mp.
52. San Marino.mp.
53. Bosnia.mp.
54. Holy See.mp. or Vatican City/
55. Czech Republic.mp.
56. Slovak Republic.mp.
57. Cyprus.mp.
58. 10 or 11 or 12 or 13 or 14 or 15 or 16 or 17 or 18 or 19 or 20 or 21 or 22 or 23 or 24 or 25 or 26 or 27 or 28 or 29 or 30 or 31 or 32 or 33 or 34 or 35 or 36 or 37 or 38 or 39 or 40 or 41 or 42 or 43 or 44 or 45 or 46 or 47 or 48 or 49 or 50 or 51 or 52 or 53 or 54 or 55 or 56 or 57
59. 1 or 2 or 3
60. 4 or 5 or 6 or 7 or 8 or 9

Supplementary Tables

Supplementary Table 1 Prior distribution of parameters for model fitting (assumed $\gamma_8 = 1$)

Parameters	Prior distribution (mean, standard deviation)
$\boldsymbol{\rho_{Austria}}$	*Normal (0.0009, 0.0017)*
$\boldsymbol{\rho_{Greece}}$	*Normal (0.000772, 0. 0017)*
$\boldsymbol{\rho_{Spain}}$	*Normal (0.0000141, 0. 0017)*
$\boldsymbol{\rho_{Romania}}$	*Normal (0.000131, 0. 0017)*
$\boldsymbol{\rho_{Cyprus}}$	*Normal (0.0000467, 0. 0017)*
$\boldsymbol{\rho_{Hungary}}$	*Normal (0.000132, 0. 0017)*
$\boldsymbol{\rho_{Italy}}$	*Normal (0.00178, 0. 0017)*
$\boldsymbol{ln(\lambda_l)}$ ***for all locations*** $\boldsymbol{l}$	*Normal (-7, 3.5)*
$\boldsymbol{\gamma_1}$ **for age group 0-9**	*Normal (0.02, 0.1)*
$\boldsymbol{\gamma_2}$ **for age group 10-19**	*Normal (0.1, 0.2)*
$\boldsymbol{\gamma_3}$ **for age group 20-29**	*Normal (0.1, 0.3)*
$\boldsymbol{\gamma_4}$ **for age group 30-39**	*Normal (0.2, 0.4)*
$\boldsymbol{\gamma_5}$ **for age group 40-49**	*Normal (0.2, 0.3)*
$\boldsymbol{\gamma_6}$ **for age group 50-59**	*Normal (0.4, 0.4)*
$\boldsymbol{\gamma_7}$ **for age group 60-69**	*Normal (0.7, 0.9)*
$\boldsymbol{\gamma_9}$ **for age group 80-89**	*Normal (1.3, 1.3)*
$\boldsymbol{\gamma_{10}}$ **for age group 90+**	*Normal (0.4, 1)*

Supplementary Table 2 Regression analysis of overall seroprevalence against serological tests used (reference category: neutralisation test) adjusted for study periods (reference group: 1958-1973).

Variable	Category	Coefficient (p-value)
**(Intercept)**	****	0.0138 (0.0233)
**Serological assay (SA)**	**HIT**	0.0138 (0.0145)
	**ELISA**	-0.00395 (0.161)
	**IFA**	0.00638 (0.153)
	**Protein microarray**	0.0596 (0.0001)
	**Unspecified IgG assay**	-0.00635 (0.736)
**Study period (t)**	**1974-1989**	0.0215 (0.0001)
	**1990-2005**	-0.00614 (0.407)
	**2006-2021**	-0.00406 (0.512)

Supplementary Figures

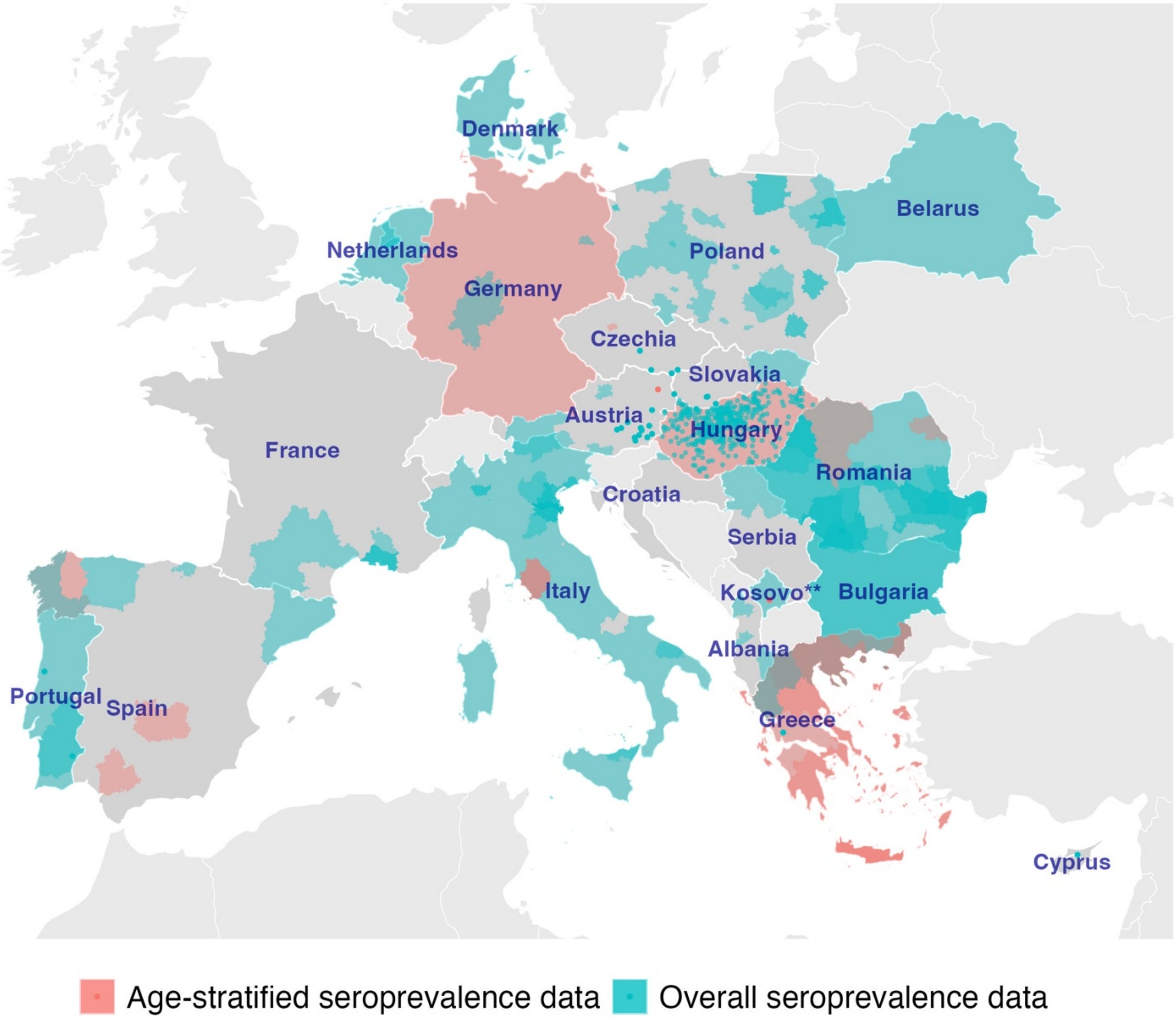

Supplementary Figure 1 Geographical distribution of regions/countries with published WNV seroprevalence studies in humans. All regions that have conducted serosurveys and reported above and including NUTS3 level are shaded in order of geographical area (from large to small). Seroprevalence studies conducted below NUTS3 level are plotted at the centroids of the survey location. Colours indicate data availability. Red shows the locations with age-stratified seroprevalence studies. Blue shows the locations where only overall seroprevalence was reported. Shaded countries indicate there are published national serosurveys. **The geographical designation is without prejudice to positions on status and is in line with United Nations Security Council Resolution 1244/99 and the International Court of Justice Opinion on the Kosovo declaration of independence.

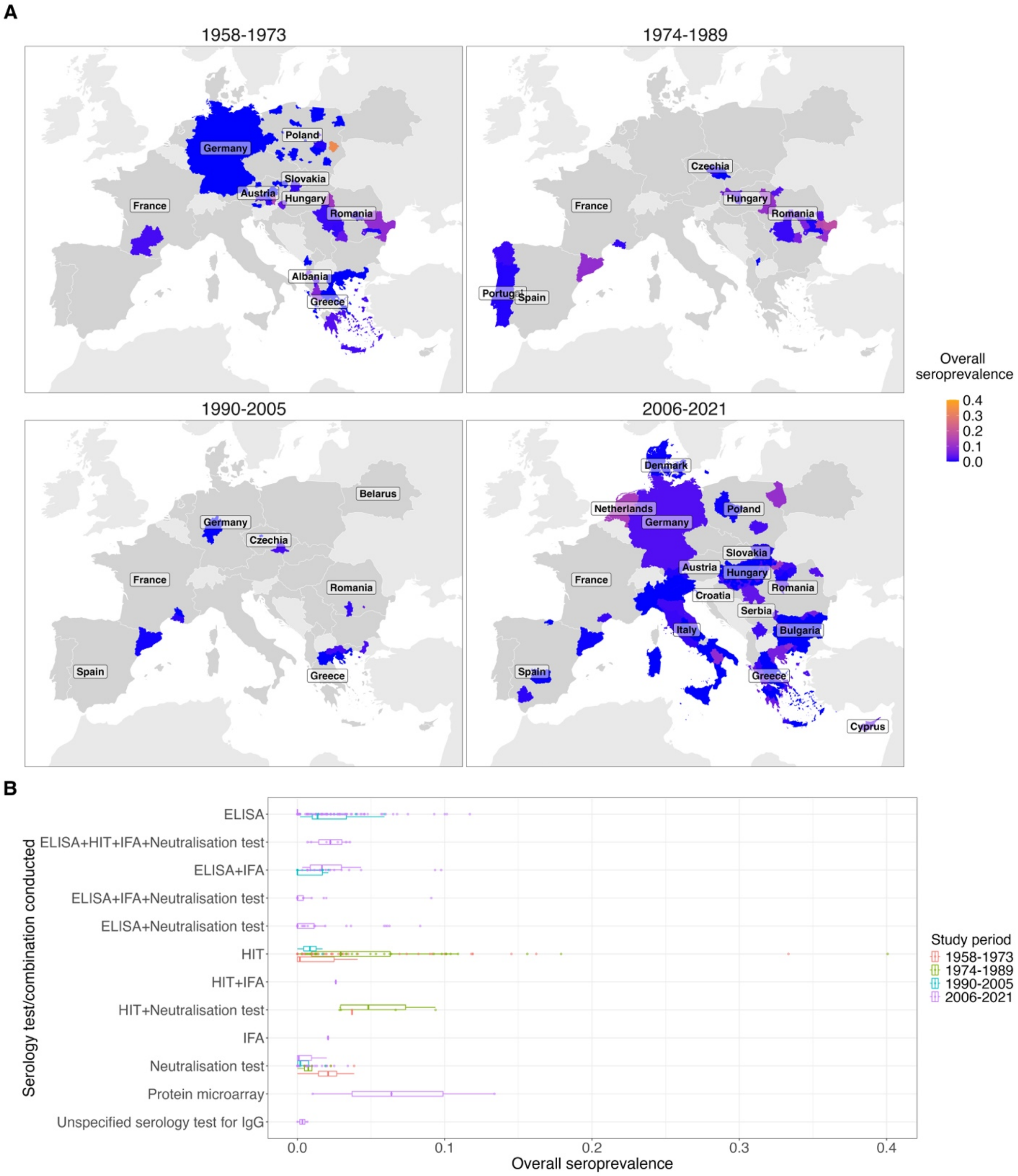

Supplementary Figure 2 Overall seroprevalence observed by time-period and serology tests. (A) Overall seroprevalence observed in eligible studies between 1958 and 2021 split into four time periods. Points identify study locations below NUTS3 level. Shaded areas represent regions at or above NUTS3 level with serosurveys. The colour indicates the magnitude of the overall seroprevalence. (B) Overall seroprevalence by serology test. The overall seroprevalence estimates are shown as points, the box shows the upper and lower quartile, and the median is indicated by the bold vertical line. The study period is indicated by colours.

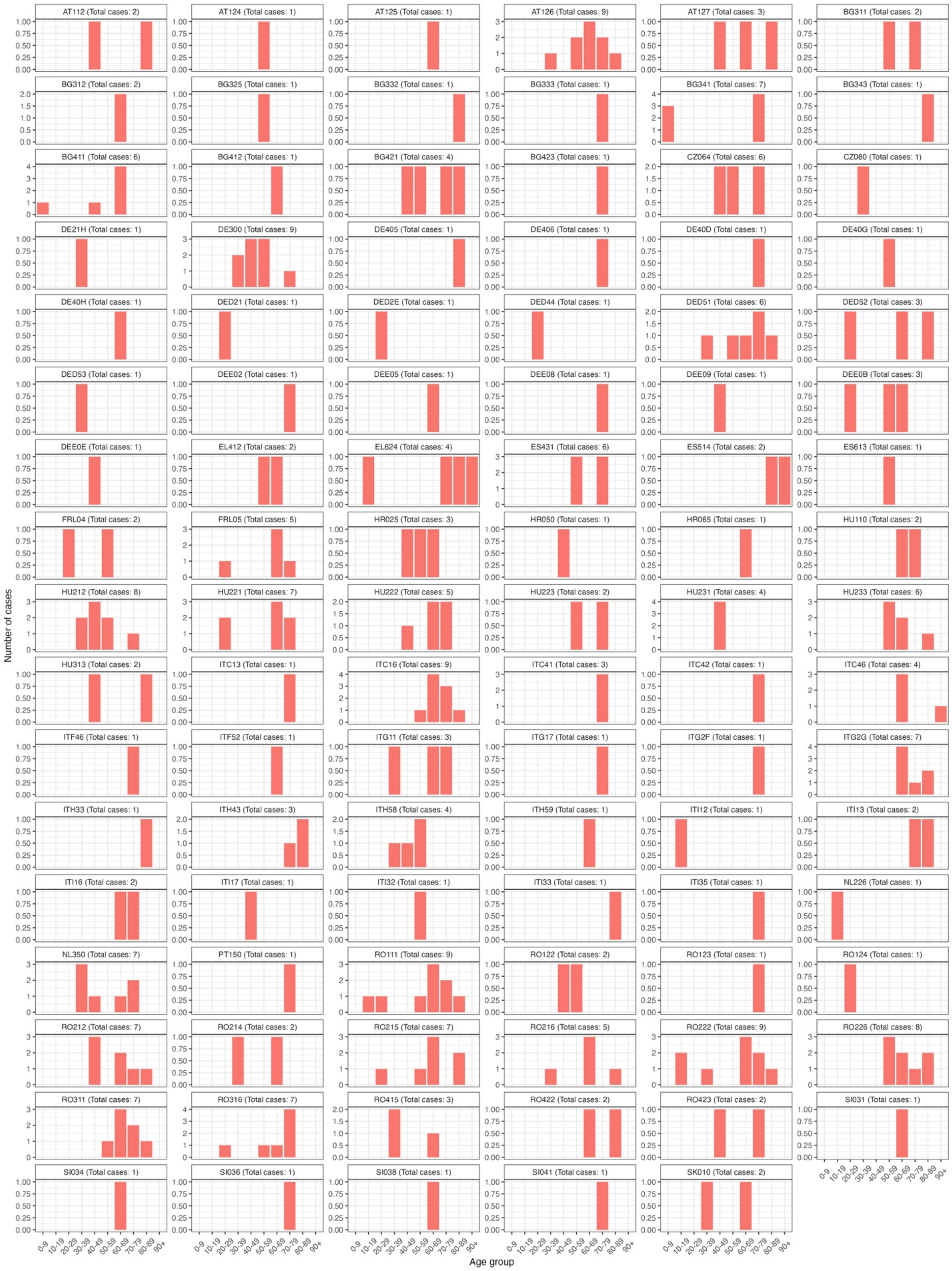

Supplementary Figure 3 Age distribution of reported cases in NUTS3 regions with <10 notified cases between 2008 and 2022 (excluded from the FOI modelling analysis). The number of cases reported by each age-group is shown and the total number of cases are indicated for each NUTS3 region with <10 cases reported during the study period.

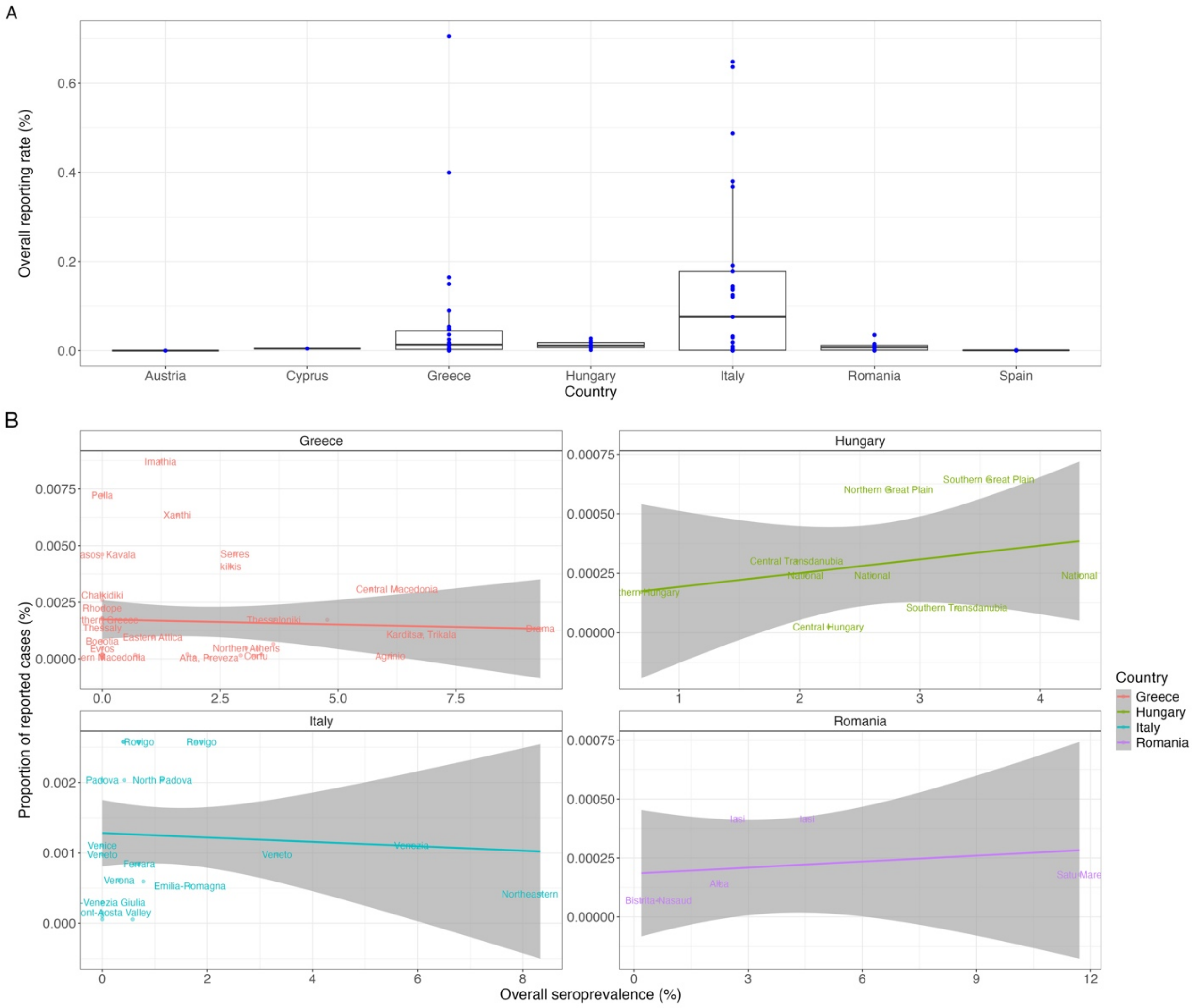

Supplementary Figure 4 Observed overall (over all age-groups) reporting rate (A) Observed (raw) overall reporting rate by country, calculated as the number of reported cases divided by the number of infections reconstructed from seroprevalence data. The blue points show the raw overall reporting rate by region, while the boxes show the median and lower and upper quartile by country. (B) Proportion of reported cases (%) vs observed overall seroprevalence (%) by country. For countries with multiple data points, the regression lines show the relationship between the proportion of reported cases and the overall seroprevalence.

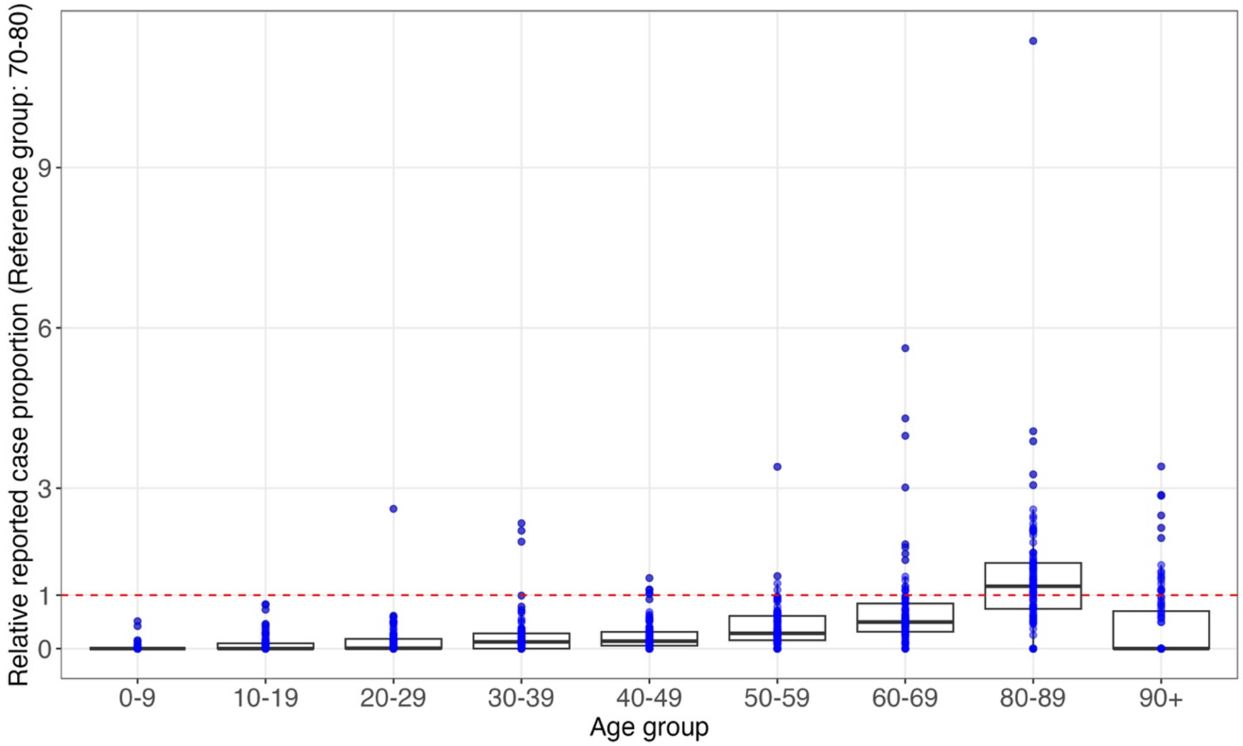

Supplementary Figure 5 Relative reported case proportion by age-group. Proportion of reported cases among the population in each age-group comparing to the reference age-group 70-80 years old. The blue points show the relative case proportion of different locations in each age-group. The box shows the upper and lower quartiles, and the bold horizontal line represents the median.

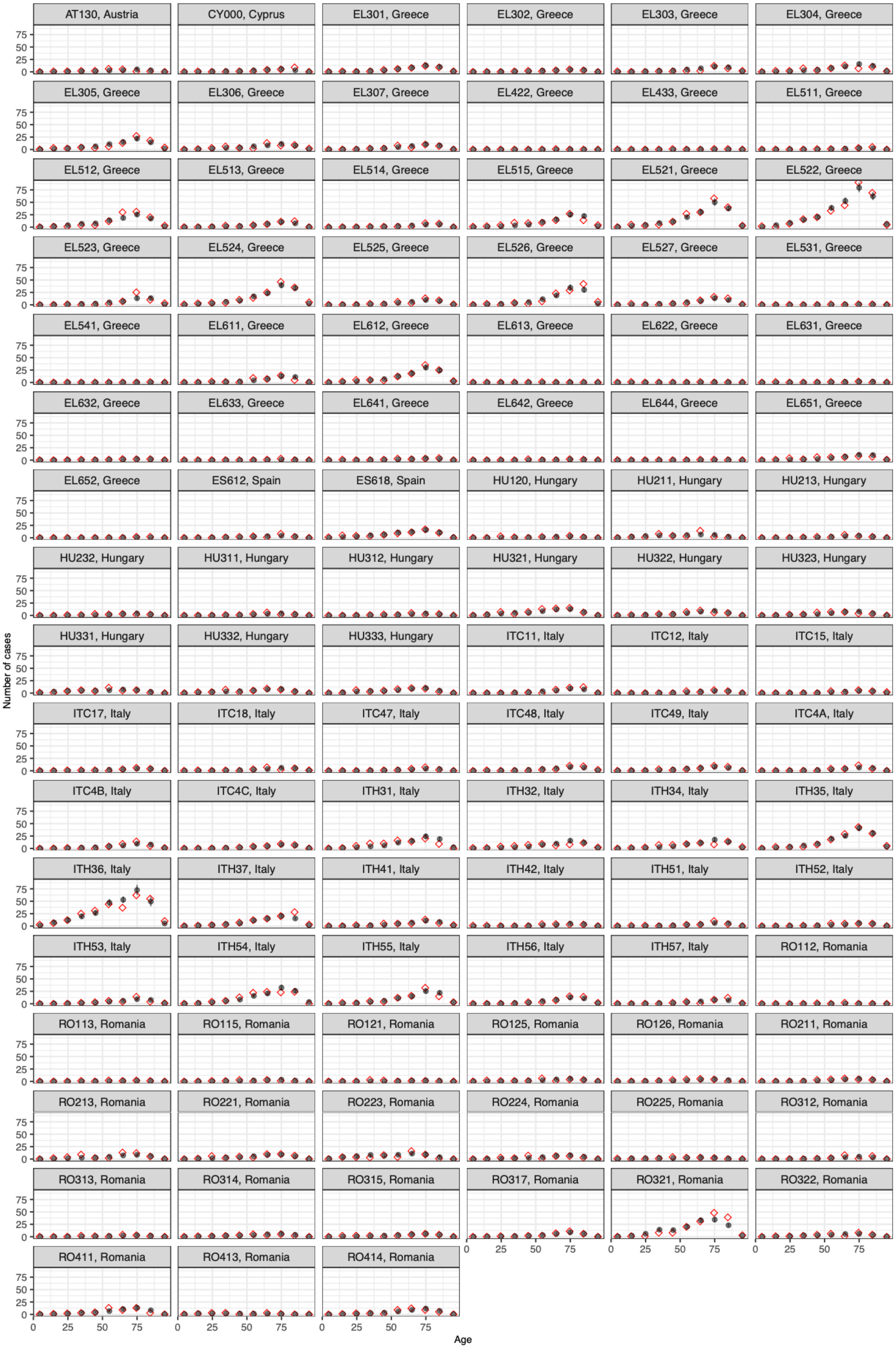
AT130, Austria
CY000, Cyprus
EL301, Greece
EL302, Greece
EL303, Greece
EL304, Greece
EL305, Greece
EL306, Greece
EL307, Greece
EL422, Greece
EL433, Greece
EL511, Greece
EL512, Greece
EL513, Greece
EL514, Greece
EL515, Greece
EL521, Greece
EL522, Greece
EL523, Greece
EL524, Greece
EL525, Greece
EL526, Greece
EL527, Greece
EL531, Greece
EL541, Greece
EL611, Greece
EL612, Greece
EL613, Greece
EL622, Greece
EL631, Greece
EL632, Greece
EL633, Greece
EL641, Greece
EL642, Greece
EL644, Greece
EL651, Greece
EL652, Greece
ES612, Spain
ES618, Spain
HU120, Hungary
HU211, Hungary
HU213, Hungary
HU232, Hungary
HU311, Hungary
HU312, Hungary
HU321, Hungary
HU322, Hungary
HU323, Hungary
HU331, Hungary
HU332, Hungary
HU333, Hungary
ITC11, Italy
ITC12, Italy
ITC15, Italy
ITC17, Italy
ITC18, Italy
ITC47, Italy
ITC48, Italy
ITC49, Italy
ITC4A, Italy
ITC4B, Italy
ITC4C, Italy
ITH31, Italy
ITH32, Italy
ITH34, Italy
ITH35, Italy
ITH36, Italy
ITH37, Italy
ITH41, Italy
ITH42, Italy
ITH51, Italy
ITH52, Italy
ITH53, Italy
ITH54, Italy
ITH55, Italy
ITH56, Italy
ITH57, Italy
RO112, Romania
RO113, Romania
RO115, Romania
RO121, Romania
RO125, Romania
RO126, Romania
RO211, Romania
RO213, Romania
RO221, Romania
RO223, Romania
RO224, Romania
RO225, Romania
RO312, Romania
RO313, Romania
RO314, Romania
RO315, Romania
RO317, Romania
RO321, Romania
RO322, Romania
RO411, Romania
RO413, Romania
RO414, Romania
Number of cases
0
25
50
75
Age

Supplementary Figure 6 Observed and estimated number and age-distribution of cumulative reported cases across the 99 NUTS3 regions reporting WNV cases between 2008 and 2022. Red points indicate the number of cases by age-group reported between 2008 and 2022. The black points and vertical line respectively indicate the estimated median reported number of cases and the 95% credible interval.

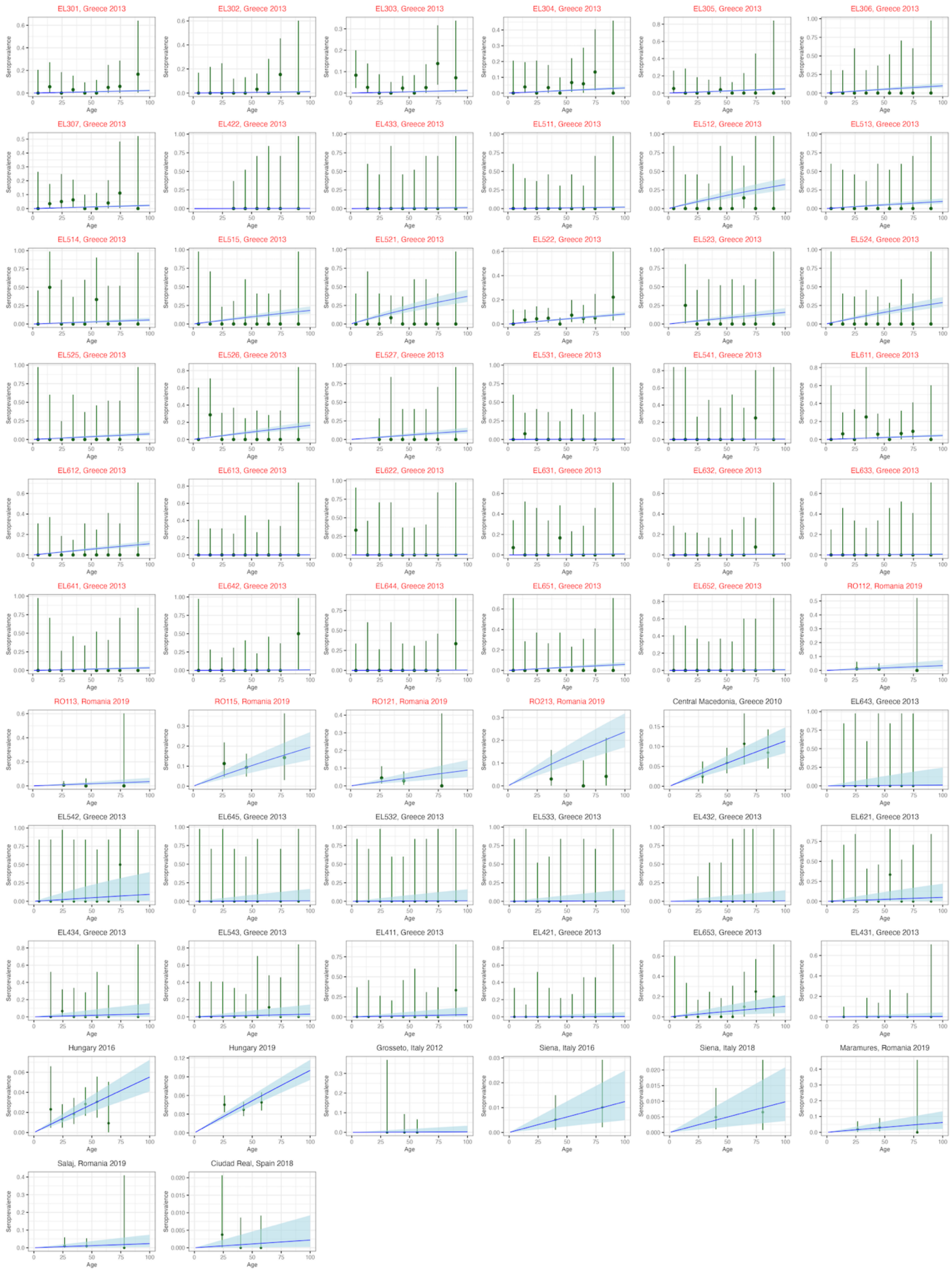

Supplementary Figure 7 Observed and estimated age-stratified seroprevalence by regions.

Regions with both age-stratified seroprevalence and case data are shown in red title while the regions with only age-stratified seroprevalence are shown in black title. Green points and vertical lines represent the observed seroprevalence and the corresponding 95% confidence interval estimated with the exact binomial method. The blue lines and shaded area show the estimated median seroprevalence and its 95% credible interval.

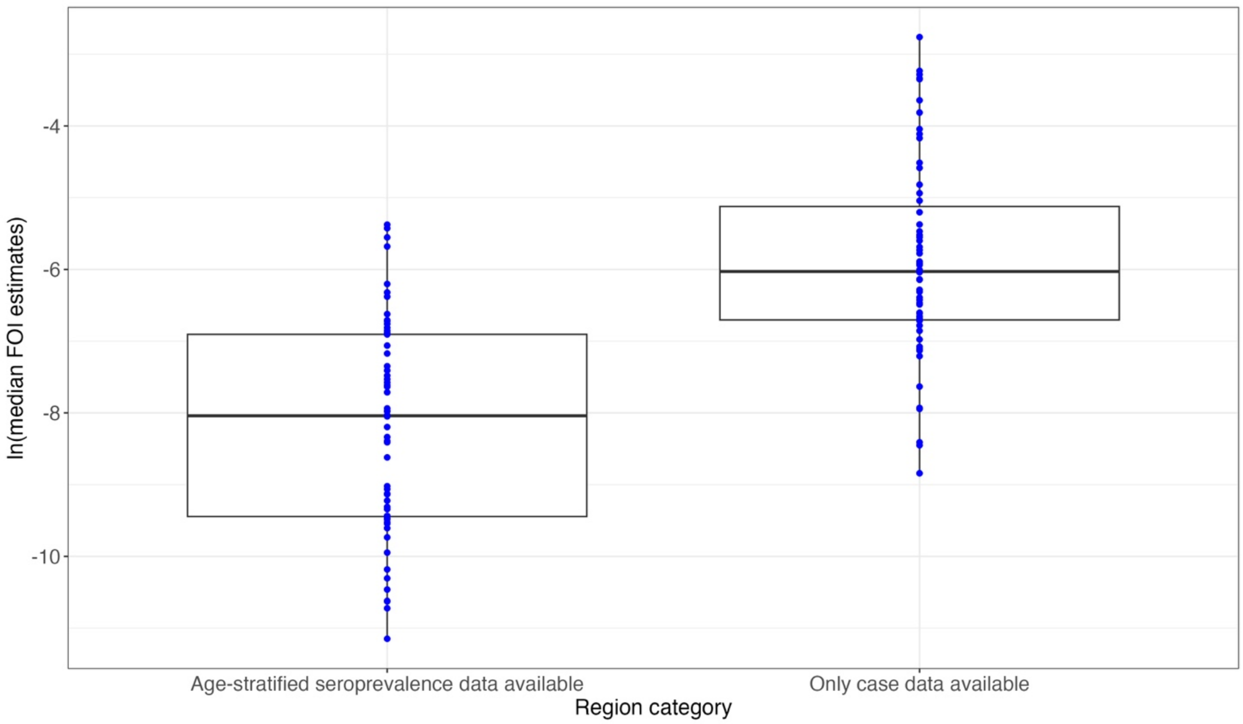

Supplementary Figure 8 Natural log-transformed (ln) median FOI estimates by region categories. The graph shows the ln(median FOI estimates) for regions with age-stratified seroprevalence data available and with only case data available. Points show the log-transformed median FOI estimates, the box shows the first and third quartile and the bold horizontal line represents the median.

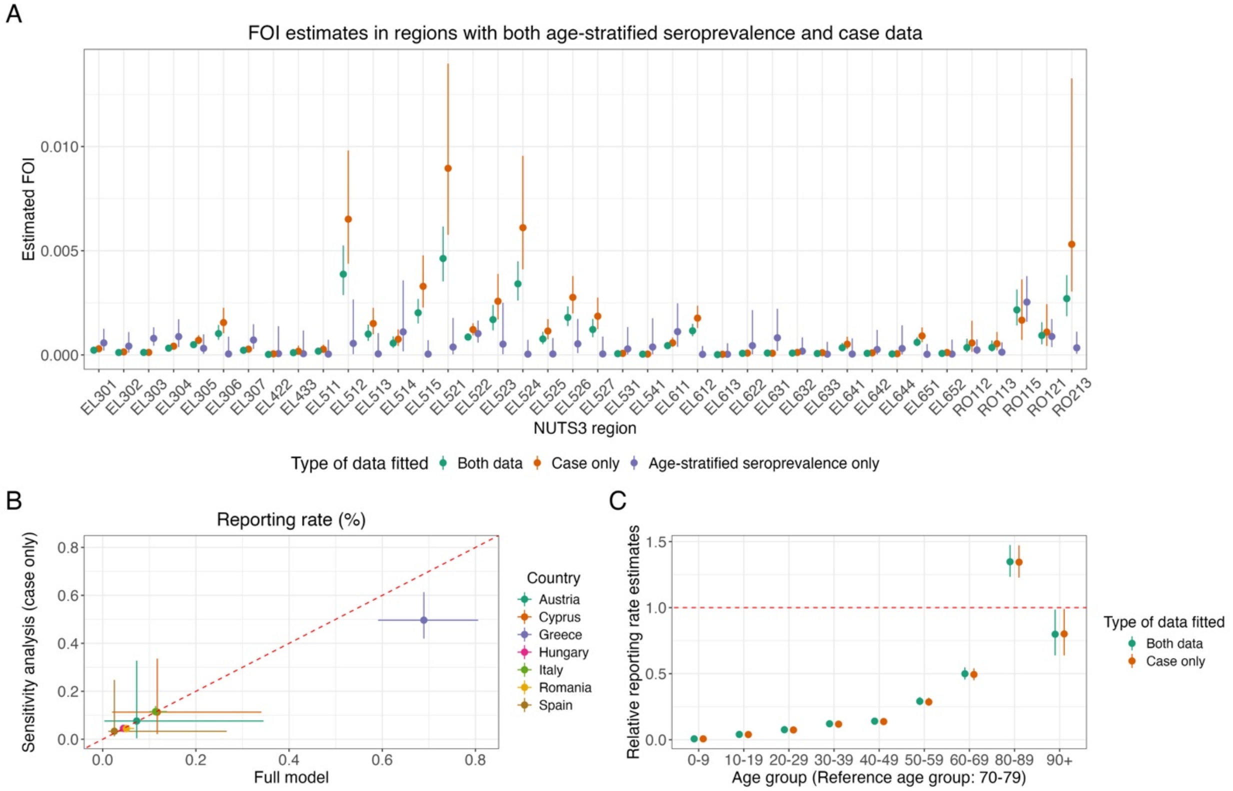

Supplementary Figure 9 Comparison of estimated parameters in the full model and in the sensitivity analyses. (A) Comparison of FOI estimates in regions with both age-stratified seroprevalence and case data obtained from the model fit to both data types (green), only case data (orange) and only seroprevalence data (purple). The points show the median estimated FOI and the error bars show the corresponding 95% credible interval. (B) Comparison of estimated reporting rate between the full model and the sensitivity analysis fitting the model to only the case data. Points show the median estimated reporting rate and the lines show the 95% credible interval. The red dashed line is the y=x line. (C) Age-dependent scaling factor of the reporting rate estimates (reference group: 70-79 years old) from the full model and the sensitivity analysis using only the case data. Green shows the estimates from the model fitted to both data types and orange shows the estimates derived from the sensitivity analysis using only case data. The point and the error bar indicate the median and 95% credible interval, respectively.

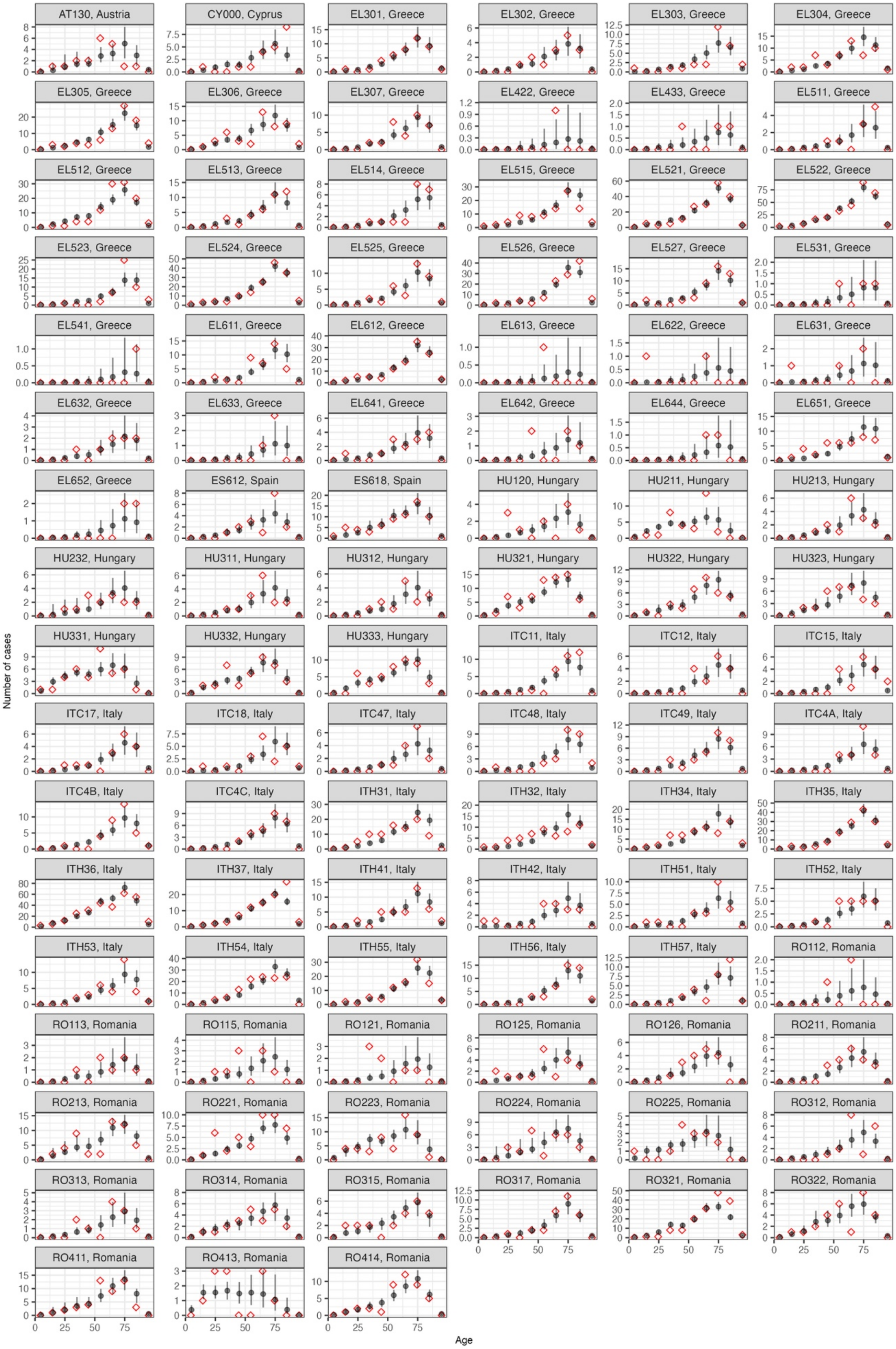
AT130, Austria
CY000, Cyprus
EL301, Greece
EL302, Greece
EL303, Greece
EL304, Greece
EL305, Greece
EL306, Greece
EL307, Greece
EL422, Greece
EL433, Greece
EL511, Greece
EL512, Greece
EL513, Greece
EL514, Greece
EL515, Greece
EL521, Greece
EL522, Greece
EL523, Greece
EL524, Greece
EL525, Greece
EL526, Greece
EL527, Greece
EL531, Greece
EL541, Greece
EL611, Greece
EL612, Greece
EL613, Greece
EL622, Greece
EL631, Greece
EL632, Greece
EL633, Greece
EL641, Greece
EL642, Greece
EL644, Greece
EL651, Greece
EL652, Greece
ES612, Spain
ES618, Spain
HU120, Hungary
HU211, Hungary
HU213, Hungary
HU232, Hungary
HU311, Hungary
HU312, Hungary
HU321, Hungary
HU322, Hungary
HU323, Hungary
HU331, Hungary
HU332, Hungary
HU333, Hungary
ITC11, Italy
ITC12, Italy
ITC15, Italy
ITC17, Italy
ITC18, Italy
ITC47, Italy
ITC48, Italy
ITC49, Italy
ITC4A, Italy
ITC4B, Italy
ITC4C, Italy
ITH31, Italy
ITH32, Italy
ITH34, Italy
ITH35, Italy
ITH36, Italy
ITH37, Italy
ITH41, Italy
ITH42, Italy
ITH51, Italy
ITH52, Italy
ITH53, Italy
ITH54, Italy
ITH55, Italy
ITH56, Italy
ITH57, Italy
RO112, Romania
RO113, Romania
RO115, Romania
RO121, Romania
RO125, Romania
RO126, Romania
RO211, Romania
RO213, Romania
RO221, Romania
RO223, Romania
RO224, Romania
RO225, Romania
RO312, Romania
RO313, Romania
RO314, Romania
RO315, Romania
RO317, Romania
RO321, Romania
RO322, Romania
RO411, Romania
RO413, Romania
RO414, Romania
Number of cases
Age

Supplementary Figure 10 Comparison of estimated and reported cases in regions with both age-stratified seroprevalence and cases data using models fitted to both data types vs using only case data. The red points show the actual number of reported cases by age-group in the NUTS3 regions. The median of the model estimates obtained on the different data are shown in green (both data types) and orange (only case data) with the error bars showing the estimated 95% credible interval.

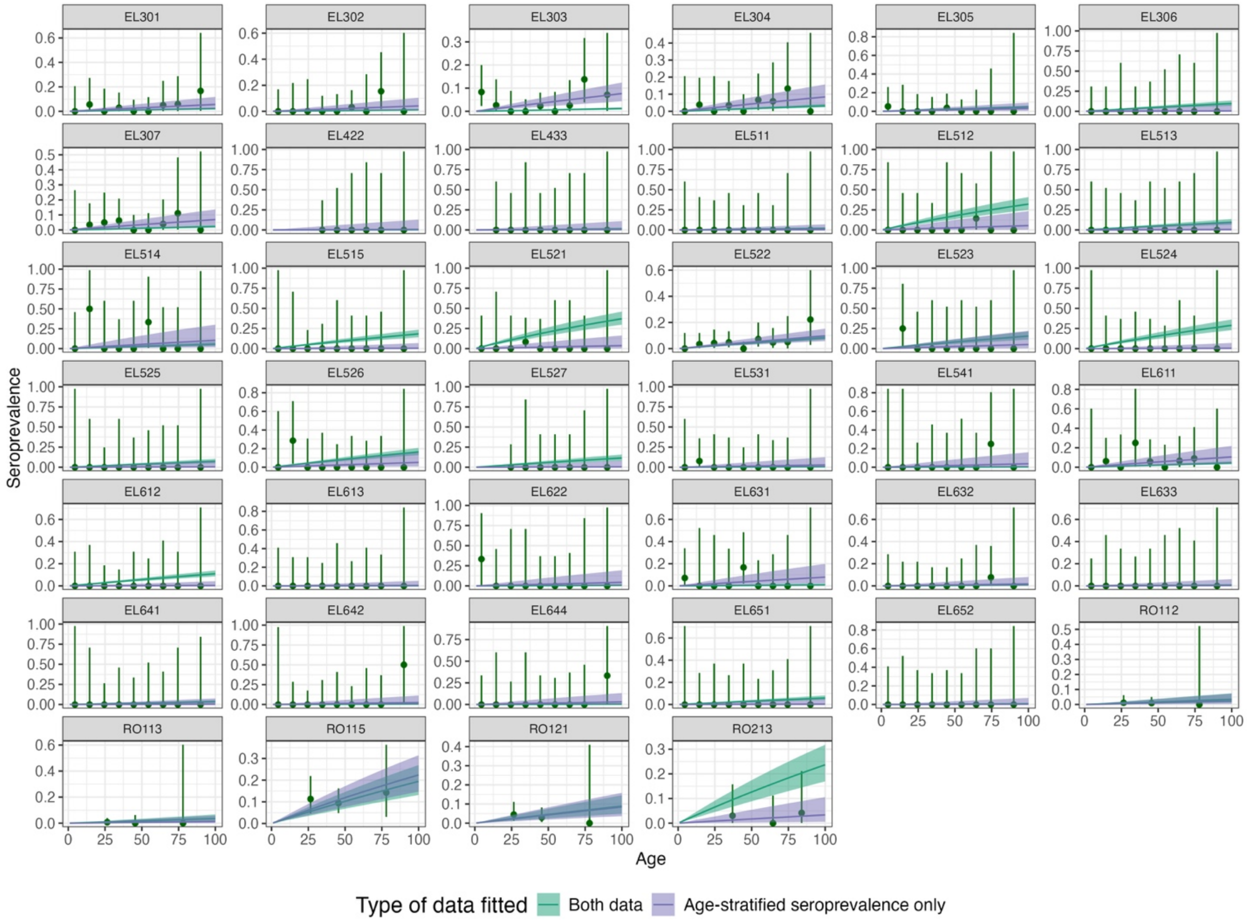

Supplementary Figure 11 Comparison of observed and estimated seroprevalence in regions with both age-stratified seroprevalence and cases data using models fitted to both data types vs using only seroprevalence data. The points and vertical lines shows the observed seroprevalence and the 95% confidence interval estimated using the exact binomial method. The median of the model estimates obtained on the different data are shown with the continuous line with the shaded area show the estimated 95% credible interval (green shows the model fitted to both data types, orange shows the model fitted to the seroprevalence data only).
